# Painful Penile Plaques: A Rare Case Report of Rectal Adenocarcinoma with Cutaneous Metastasis to the Penis

**DOI:** 10.7759/cureus.5095

**Published:** 2019-07-08

**Authors:** Syed W Ahmad, Robert P Daze, Sarah Arvaneh, Said Awad

**Affiliations:** 1 Medical Education, Nova Southeastern University, Dr. Kiran C. Patel College of Osteopathic Medicine, Fort Lauderdale, USA; 2 Dermatology, Largo Medical Center, Largo, USA; 3 Internal Medicine, Largo Medical Center, Largo, USA

**Keywords:** rectal adenocarcinoma, penile metastasis, secondary malignancy, penile cancer, cutaneous metastasis, penis, metastatic rectal adenocarcinoma, penile neoplasm

## Abstract

Despite the rich vascularization of the penis and its close proximity to other pelvic organs, cutaneous manifestations of metastatic disease to the penis are an uncommon occurrence. Penile lesions suspected for malignancy should alert clinicians to differentiate between primary and secondary tumors. While the majority of metastatic malignancies arise from the genitourinary tract, we present a unique case report of a 51-year-old male with penile metastasis of primary rectal adenocarcinoma. A thorough diagnostic evaluation was performed including imaging studies, colonoscopy, as well as penile biopsies with associated immunohistochemistry panel. The patient was diagnosed with penile metastases secondary to invasive rectal adenocarcinoma. Due to the aggressive nature of the patient’s presentation, systemic chemotherapy was initiated for palliative measures as the patient declined any radical surgical intervention.

## Introduction

Cutaneous metastases to the penis and scrotum arising from primary colorectal adenocarcinoma are extremely uncommon. Squamous cell carcinomas are the most common type of primary penile malignancy [1]. Primary metastatic tumors to the penis are usually from the genitourinary organs, such as the bladder and prostate, accounting for approximately 69% of reported cases [2]. The gastrointestinal system is the second most common site of primary tumors that metastasize to the penis [3]. A review of metastatic tumors to penis has shown that primary tumors of the rectum and sigmoid colon account for just 12% of the entirety of all such cases [4]. Clinical evidence of penile metastases is an ominous sign that is associated with widely disseminated disease, yielding a poor prognosis regardless of the primary tumor site or treatment [5]. Our case is unique in that it appears to be only the second of its kind reported in the literature in which the penile metastases were the initial reason for the patient’s presentation to the hospital. We report a case of penile cutaneous metastases secondary to a primary rectal adenocarcinoma and discuss the clinical differential, diagnostics, and prognostic management of penile metastases. 

## Case presentation

**Figure 1:**
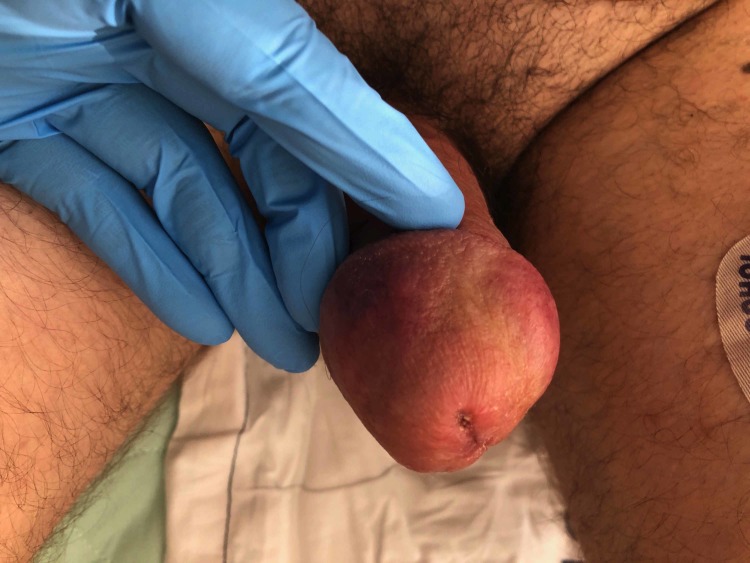
Well-demarcated, indurated, erythematous plaques of the penile corona

**Figure 2:**
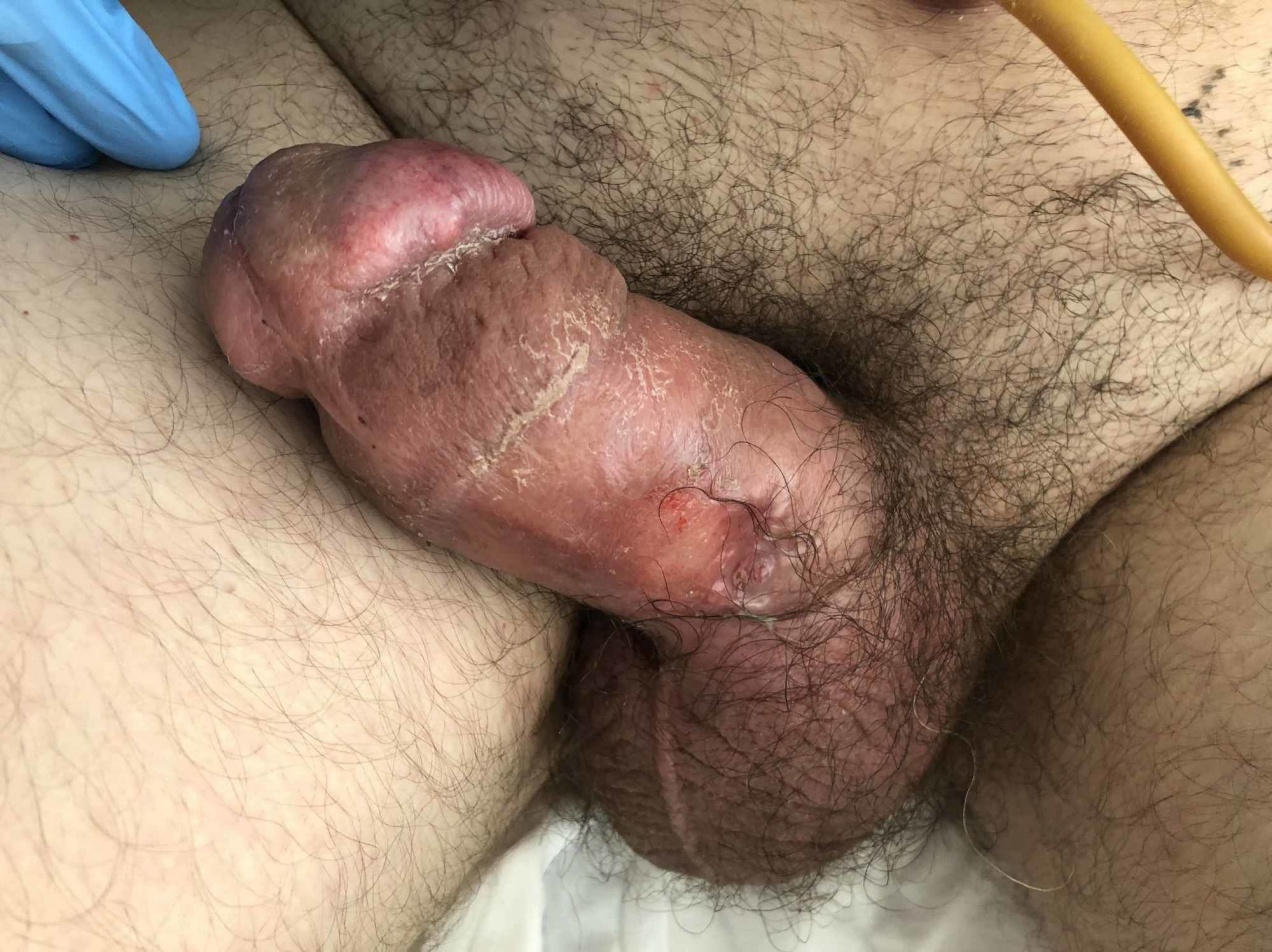
Indurated, erythematous plaque of the corona with associated swelling of the penile shaft

We report a case of a 51-year-old Caucasian male who presented to the emergency department with severe groin pain for the past five days. The patient also complained of multiple, painful, subcutaneous nodules to the penile shaft and scrotum. Physical examination demonstrated two well-demarcated, indurated, painful plaques involving the glans of the penis (Figure 1). The penile shaft was diffusely swollen (Figure 2). A markedly painful, subcutaneous nodule was also present in the perineum. Patient denied symptoms of discharge, dysuria, hematuria, proctalgia, and constipation. 

**Figure 3:**
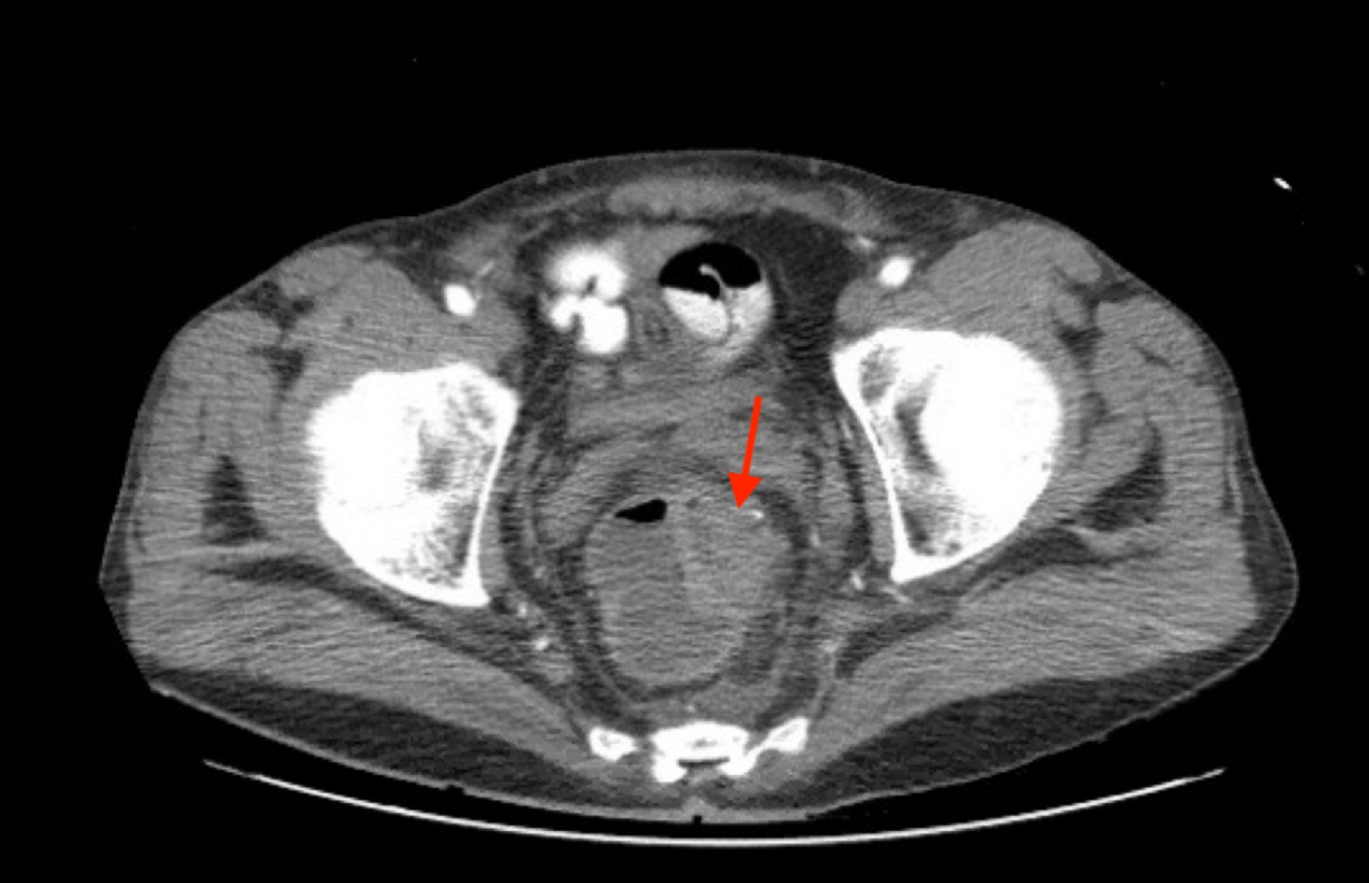
3-cm asymmetrical rectal mass, highly concerning for malignancy

**Figure 4:**
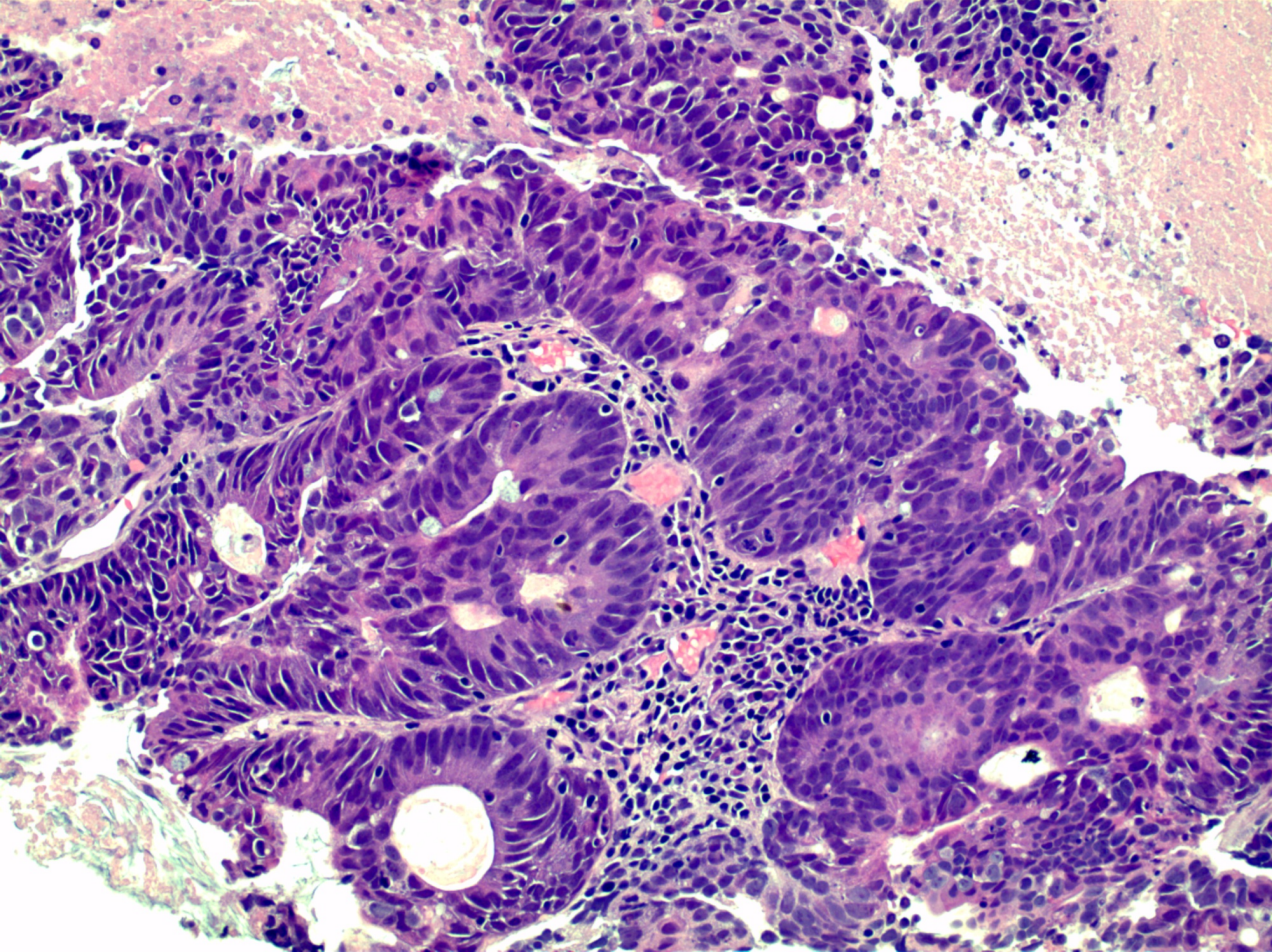
Penile mass demonstrating high-grade invasive adenocarcinoma (Hematoxylin and eosin, high power)

**Figure 5:**
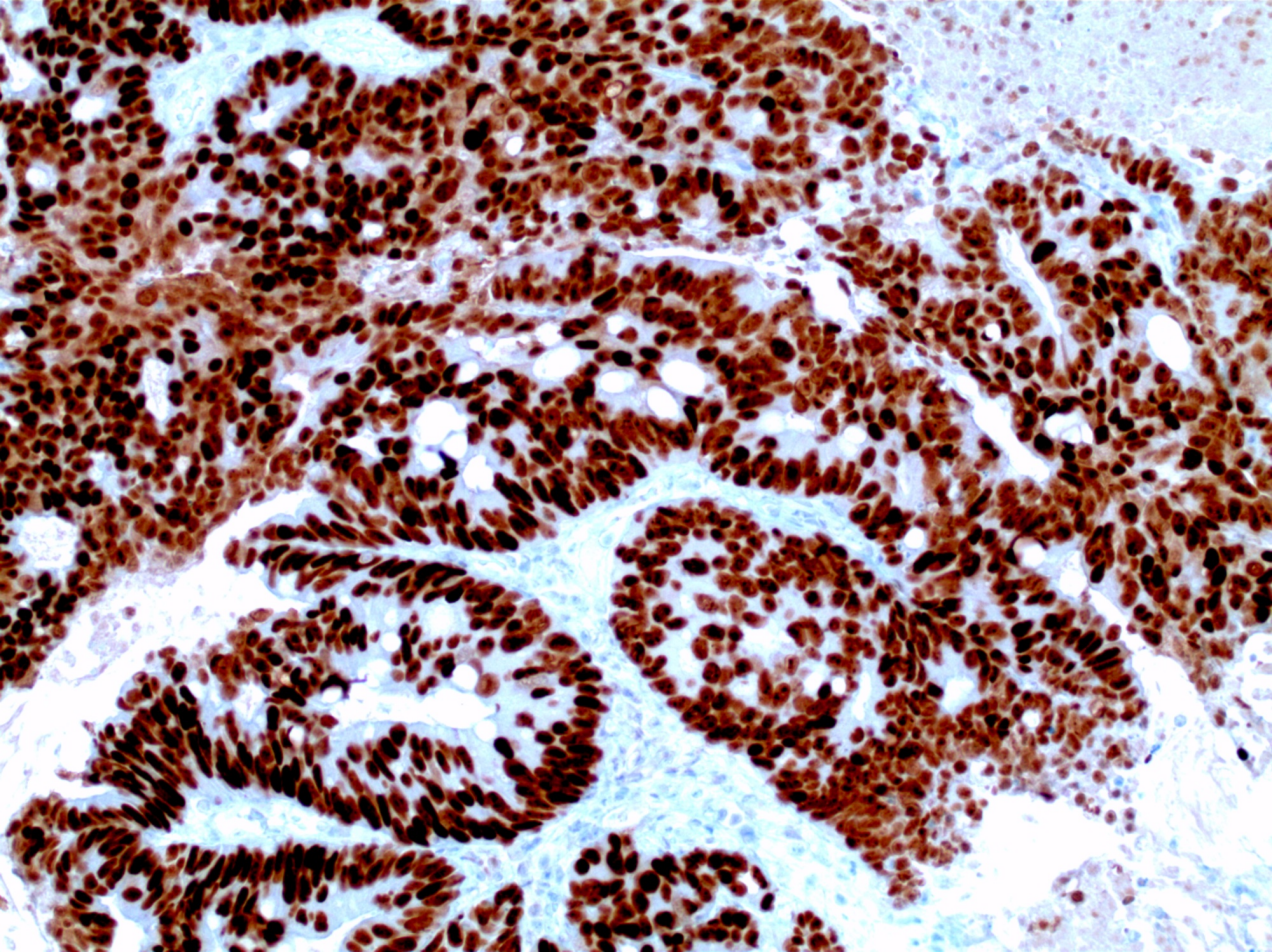
Penile mass with immunohistochemical staining demonstrating positive staining CDX-2, confirming gastrointestinal adenocarcinoma (high power)

**Figure 6:**
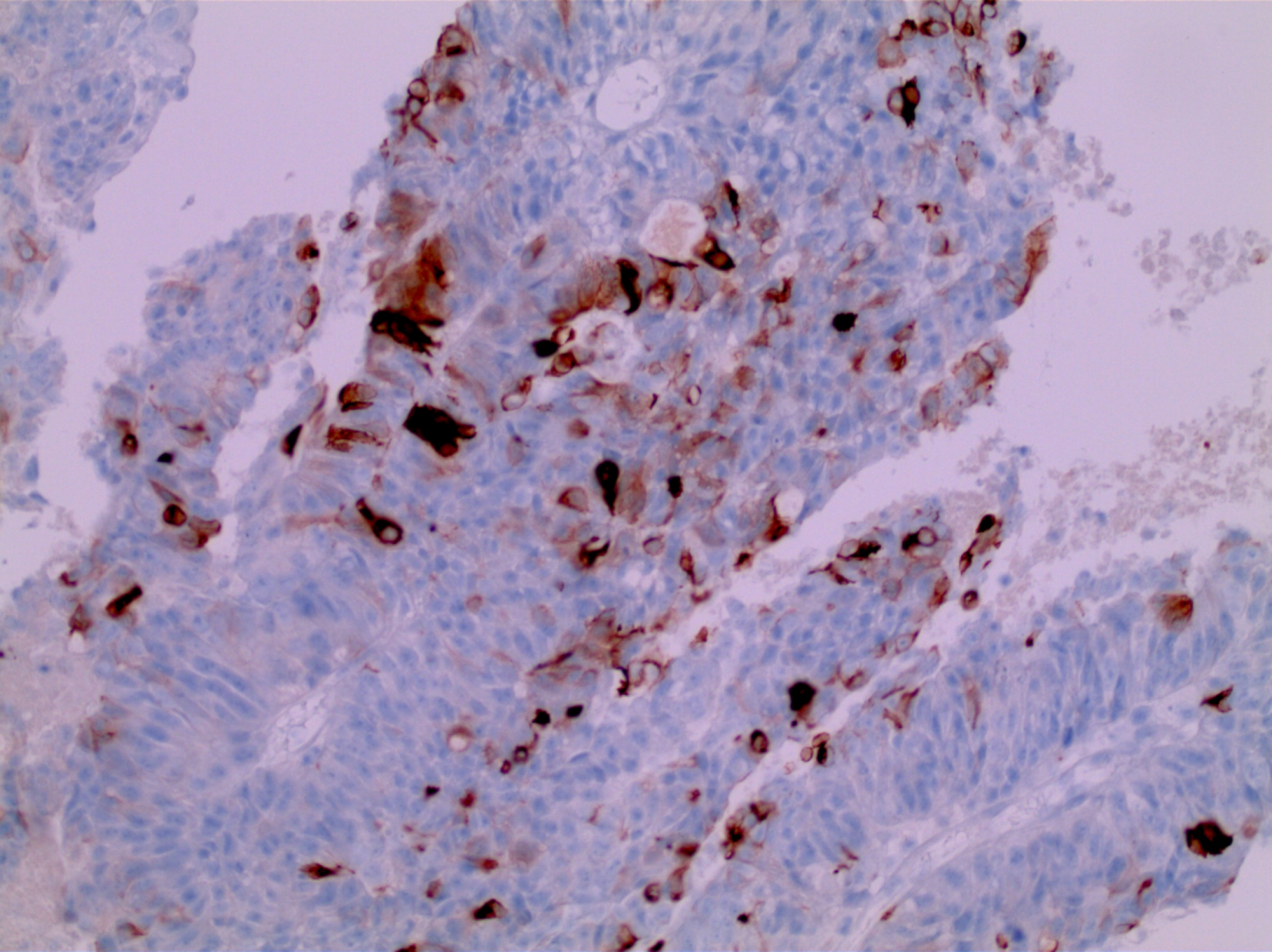
Penile mass with immunohistochemical staining demonstrating positive staining for CK20, confirming gastrointestinal adenocarcinoma (high power)

Ultrasound of the testicles showed normal appearance of the testicles with no evidence of any intratesticular mass. Computed tomography of the abdomen and pelvis revealed two rim-enhancing hypodense ovoid shaped lesions in the central and left lateral aspect of the perineum, inferiorly and left laterally adjacent to the root of the penis. Incidentally, it also revealed asymmetric wall thickening of the rectum highly concerning for malignancy (Figure 3), and likely metastatic masses of the spleen and left hepatic lobe. Carcinoembryonic antigen (CEA) level was elevated at 21.5 ng/mL (reference range 0.0-5.0 ng/mL). Alpha fetoprotein (AFP) level was 2.4 ng/mL (reference range 0.0-8.0 ng/mL) and carbohydrate antigen (CA 19-9) was 21.5 units/mL (reference range 0.0-30.9 units/mL), were both within normal reference range. Serological studies for gonorrhea, chlamydia and human immunodeficiency virus (HIV) were unremarkable. Cystoscopy revealed an extrinsic urethral mass with obstructing erosion into the urethra. Additionally, in order to confirm the diagnosis, both a cutaneous and transurethral biopsy were performed. Hematoxylin and eosin staining of the specimen demonstrated a high-grade invasive adenocarcinoma (Figure 4). Immunohistochemical staining of the penile mass and paraurethral mass were positive for Cytokeratin 20 (CK20) and Caudal type homeobox 2 (CDX-2), which both are used in identifying gastrointestinal adenocarcinoma (Figures 5-6). Furthermore, it was negative for Cytokeratin 7 (CK7) and GATA Binding Protein 3 (GATA-3), which are utilized to rule out urothelial carcinomas. And finally it was negative for Paired box gene 8 (PAX-8), excluding renal cell carcinoma. Follow up colonoscopy demonstrated a 5-cm ulcerated necrotic mass in the rectum. The biopsy demonstrated moderately differentiated rectal adenocarcinoma. The above clinical and histopathological findings were consistent with high grade, invasive adenocarcinoma favoring a rectal primary.

Furthermore, due to the urethral obstruction, a suprapubic catheter was placed. The patient was offered a palliative penectomy due to intractable groin pain but declined. Subsequently, he was referred to outpatient oncology for the initiation of palliative systemic chemotherapy including FOLFOX (5-fluorouracil, leucovorin, oxaliplatin) and bevacizumab. Also, he was unable to complete a second chemotherapy administration due to a second hospitalization for intractable groin pain and ulceration with purulent drainage from the metastatic nodules, two months after his initial presentation. Wound cultures were methicillin-resistant *Staphylococcus aureus *(MRSA) positive, and urine cultures were positive for *Enterococcus spp*. and MRSA. A penile evacuation and incision and drainage of the abscesses were performed in addition to a replacement of the suprapubic catheter. The patient was discharged from the hospital and currently awaiting reinitiation of his chemotherapy pending clearance from the aforementioned infections.

## Discussion

Despite the rich vascularization of the penis, cutaneous manifestations of metastatic disease to the penis are a rare phenomenon. While the majority of primary malignancies arise from the genitourinary tract, we present a unique case of penile metastasis of primary rectal adenocarcinoma. 

Routes of metastases are not fully understood, however most authors postulate several pathways: retrograde venous route, retrograde lymphatic route, arterial spread, direct extension, and iatrogenic secondary to instrument implantation [3-4,6-7]. Most of the literature delineates retrograde venous transportation as the main mechanism involved in secondary penile cutaneous malignancies [3-4,6,8-9]. The dorsal venous system of the penis and the venous system of the pelvic organs are in direct communication [4,8]. As such, with a tumor causing proximal obstruction, retrograde flow allows for the malignant cells to travel back to the corpora cavernosa and the glans of the penis [4,6]. Retrograde lymphatic flow is similar in mechanism to the venous system, with the iliac nodes providing a convergence between the two systems [4]. Arterial spread, while exceedingly rare, is secondary to tumor extension into the arterial pathways [4,8]. Direct extension is likely to occur from the tumor spreading along perineal planes to reach the penis [10]. Finally, tumor metastasis secondary to implantation involves physical inoculation via surgical instruments [4]. 

The most common chief complaint is malignant priapism [4-6,11]. With penile metastases other clinical manifestations include urinary retention, hematuria, nodules, ulceration, generalized swelling, and edema [3,6,9]. Our case is unique in that the patient did not present with any gastrointestinal symptoms related to his underlying rectal adenocarcinoma (i.e. hematochezia, melena, change of bowel habits, proctalgia, and tenesmus). 

With cutaneous metastasis to the penis being a rare entity, an astute clinical differential should be thoroughly considered when evaluating penile lesions of unknown etiology. Pre-malignant and primary malignancies (Bowen disease, Erythroplasia of Queyrat, verrucous carcinoma, squamous cell carcinoma, melanoma, Extramammary Paget’s disease), infectious causes (tuberculosis, chancroid, syphilis), and non-malignant lesions (Peyronie’s disease, non-tumoral priapism) should be investigated through a targeted history and physical and subsequent diagnostics [3-4,6]. Accurate diagnosis should include a biopsy with immunohistochemical stains to distinguish between primary and secondary tumors of the penis [3-5,9-10]. As a non-invasive imaging modality, magnetic resonance imaging remains the preferred imaging to evaluate the extent of metastases [3,5,8-10]. Ultrasonography is user dependent and computed tomography in only a single plane limits the diagnostics [4].

The literature reveals that most patients with penile metastases have widespread disease at the time of presentation [2-9]. The prognosis remains very poor with an 80% mortality within six months regardless of treatment option [5-6]. No single treatment has been standardized or superior to another. Treatment options include surgical excision, total penectomy, radiotherapy, chemotherapy, and symptomatic management [4-5,7-11]. Total penectomy is the only treatment option to afford a longer-term survival in patients with localized disease, extending mean survival to 11 months [10]. With the short life expectancy, treatment is aimed at palliative efforts.

## Conclusions

In conclusion, cutaneous metastases to the penis from rectal adenocarcinoma are a rare entity, as the majority of secondary cutaneous penile malignancies arise from the genitourinary tract. Despite its paucity, clinicians should approach penile metastases with appropriate diagnostic and therapeutic modalities. It is important to recognize this unusual presentation to initiate timely palliative and supportive care measures.
